# Supplementary material for: MetaRibo-Seq measures translation in microbiomes
Source: Nat Commun. 2020 Jun 29;11:3268. doi: 10.1038/s41467-020-17081-z (PMC7324362; doi:10.1038/s41467-020-17081-z)
Supplement: Supplementary file 10 — Supplementary Data 7 [file 41467_2020_17081_MOESM10_ESM.zip › File2/Confidence_VeryHigh_Taxonomy/59935_out.krona.html]

Javascript must be enabled to view this page.

members
magnitude
magnitudeUnassigned
count
unassigned
taxon
rank

59935\_out

10

superkingdom
2
10

phylum
1239
10

10
class
186801

1
186802

SRS143780\_contig\_number\_contig-100\_14419.48401
order
10

31979
family
9

genus
580596
9

2292003

SRS149879\_contig\_number\_contig-100\_1823.152735SRS893270\_contig\_number\_21065
species
2

species

SRS893378\_contig\_number\_1615
2292197
1


SRS015578\_contig\_number\_30180SRS097889\_contig\_number\_11053SRS148159\_contig\_number\_11722SRS149181\_contig\_number\_2575
species
1193532
4


SRS014235\_contig\_number\_contig-100\_29686.121263SRS015854\_contig\_number\_15361
2292293
species
2
